# Cerebrospinal Fluid Cytokines and Neurodegeneration‐Associated Proteins in Parkinson's Disease

**DOI:** 10.1002/mds.28015

**Published:** 2020-03-04

**Authors:** Ruwani S. Wijeyekoon, Sarah F. Moore, Krista Farrell, David P. Breen, Roger A. Barker, Caroline H. Williams‐Gray

**Affiliations:** ^1^ John van Geest Centre for Brain Repair, Department of Clinical Neurosciences University of Cambridge Cambridge United Kingdom; ^2^ University of Exeter Medical School, University of Exeter Exeter United Kingdom; ^3^ Centre for Clinical Brain Sciences University of Edinburgh Edinburgh Scotland United Kingdom; ^4^ Anne Rowling Regenerative Neurology Clinic, University of Edinburgh Edinburgh Scotland United Kingdom; ^5^ Usher Institute of Population Health Sciences and Informatics, University of Edinburgh Edinburgh Scotland United Kingdom; ^6^ Wellcome Trust‐MRC Cambridge Stem Cell Institute, University of Cambridge Cambridge United Kingdom

**Keywords:** alpha‐synuclein, cerebrospinal fluid, cytokine, Parkinson's disease, tau

## Abstract

**Introduction:**

Immune markers are altered in Parkinson's disease (PD), but relationships between cerebrospinal fluid (CSF) and plasma cytokines and associations with neurodegeneration‐associated proteins remain unclear.

**Methods:**

CSF and plasma samples and demographic/clinical measures were obtained from 35 PD patients. CSF samples were analyzed for cytokines (together with plasma) and for α‐synuclein, amyloid β(1‐42) peptide, total tau, and phospho(Thr231)‐tau.

**Results:**

There were no CSF–plasma cytokine correlations. Interleukin (IL)‐8 was higher and interferon‐γ, IL‐10, and tumor necrosis factor–α were lower in CSF versus plasma. In CSF, total tau correlated positively with IL‐8 and IL‐1β, whereas α‐synuclein correlated positively with amyloid β(1‐42) and negatively with semantic fluency (a known marker of PD dementia risk).

**Discussion:**

CSF and peripheral cytokine profiles in PD are not closely related. Associations between CSF IL‐8 and IL‐1β and tau suggest that CSF inflammatory changes may relate to tau pathology within PD. CSF α‐synuclein/amyloid β may reflect the risk of developing PD dementia. © 2020 The Authors. *Movement Disorders* published by Wiley Periodicals, Inc. on behalf of International Parkinson and Movement Disorder Society.

Parkinson's disease (PD) is associated with central and peripheral immune changes.^1^ However, the relationships between these changes and central neurodegeneration are unclear. The cerebrospinal fluid (CSF) is in close contact with the central nervous system (CNS), and studying CSF immune markers and neurodegeneration‐associated proteins alongside paired plasma immune markers may provide additional insights into these relationships in PD.

The key neurodegeneration‐associated protein involved in PD is α‐synuclein, with multiple factors leading to abnormal aggregation and pathology. CSF total α‐synuclein concentration is generally decreased in PD when compared with controls,^2^ possibly reflecting intracellular accumulation/aggregation. PD patients with cognitive impairment and dementia additionally have decreased CSF amyloid β and increased total tau levels.^3^

Increased levels of inflammatory cytokines (eg, interleukin [IL]‐1β, IL‐6, IL‐18, and tumor necrosis factor [TNF]‐α) have been detected in PD CSF when compared with controls,^4^, ^5^, ^6^ and many studies have reported elevated levels of inflammatory cytokines in the serum/plasma of PD patients when compared with controls.^7^ A more proinflammatory serum cytokine profile has further been associated with more rapid disease progression in early PD,^8^ and although peripheral cytokine transport across the blood–brain barrier, with mediation of microglial activation and neuronal damage, could impact on the disease course, a consistent relationship between peripheral and central cytokine levels has not been demonstrated in PD.

The relationships between CSF cytokine changes and neurodegeneration‐associated protein levels are largely unknown. Currently, limited studies have investigated this in PD,^9^ and no studies have investigated CSF α‐synuclein, amyloid β, total tau, and phospho‐tau alongside CSF cytokines in a single PD cohort.

This study aimed to examine the relationships between central and peripheral cytokine levels as well as CSF cytokine and neurodegeneration‐associated protein associations in a well‐characterized, moderate‐stage PD cohort, to provide further insight into the drivers of central immune activation in PD.

## Methods

### Patient Recruitment

Ethical approval was obtained from the Cambridgeshire‐2 Research Ethics Committee (08/H0308/331). Patients were recruited from the PD Research Clinic at the John van Geest Centre for Brain Repair in Cambridge. Following screening for contraindications to lumbar puncture, written informed consent was obtained. Clinical data gathered included demographic, medical/drug history, Movement Disorder Society–Unified Parkinson's Disease Rating Scale (MDS‐UPDRS), Addenbrooke's Cognitive Examination–Revised (ACE‐R), semantic fluency (predictive of dementia in PD),^10^ and Beck Depression Inventory scores.

### Sample Collection and Processing

Lumbar punctures were performed in the left lateral position at the L3/4 or 4/5 space using aseptic technique, 1% lignocaine as local anaesthetic and a 22G spinal needle; ~2 to 5 mL of CSF was collected. A subset of patients (n = 22) had concurrent ethylenediamine tetraacetic acid (EDTA) venous blood sampling.

CSF samples were centrifuged at 3000*g* for 15 minutes. Supernatant was stored in ~500 uL aliquots at −80°C. Plasma was extracted from blood by centrifugation at 2000 rpm (~670*g*) for 15 minutes and stored at −80°C.

### Cytokine and Protein Analysis

Samples were analyzed using the Meso Scale Diagnostics (Rockville, MD) electrochemiluminescence platform. Assays were performed in duplicate, at 1:2 dilution, and according to the manufacturer's instructions (https://www.mesoscale.com/): V‐PLEX proinflammatory panel‐1 cytokines (interferon [IFN]‐γ, IL‐1β, IL‐2, IL‐4, IL‐6, IL‐8, IL‐10, IL‐12p70, IL‐13, TNF‐α) in CSF and plasma, and α‐synuclein, phospho(Thr231)‐tau, total tau, and amyloid β(1‐42) in CSF. Plates were read using the Meso Scale Diagnostics SECTOR Imager. Data were processed using Meso Scale Diagnostics Discovery Workbench software.

### Statistical Analysis

Data were analyzed using IBM (Armonk, NY) Statistical Package for the Social Sciences (SPSS) version 25 and GraphPad (San Diego, CA) Prism 7. Cytokine and protein variables within the CSF and plasma with assay‐detected values in >75% of participants were included in the analysis. Log_10_ transformation was performed because of the nonparametric distribution of variables.

Plasma and CSF cytokine profiles were compared using a repeated‐measures analysis of variance and paired *t* tests, with Bonferroni correction for multiple testing as appropriate. Bivariate correlations were assessed between CSF cytokines and neurodegeneration‐associated proteins and between CSF markers and clinical measures. Variables with uncorrected significant correlations (*P* < 0.05) were included in linear regression analyses with adjustments for relevant confounders.

## Results

### Participant Demographics

Demographic and clinical measures in the PD cohort (n = 35) were expressed as mean (standard deviation) or percentage: age 65.4 (7.6) years, sex 48.6% male, years of education 18.7 (3.9), disease duration 5.4 (5.6) years, Movement Disorder Society–Unified Parkinson's Disease Rating Scale part III (*on* treatment) 31.0 (12.1), ACE‐R score 90.3 (9.4), Beck Depression Inventory score 9.1 (7.7), and semantic fluency score 23.9 (8.4).

CSF samples from all patients and paired plasma samples from 22 patients were available for analysis.

### CSF and Plasma Cytokines

The cytokines IFN‐γ, IL‐1β, IL‐2, IL‐6, IL‐8, IL‐10, IL‐12p70, and TNF‐α in CSF and IFN‐γ, IL‐6, IL‐8, IL‐10, and TNF‐α in plasma had measurable values in >75% of the analyzed samples and were used for further analysis.

**Figure 1:**
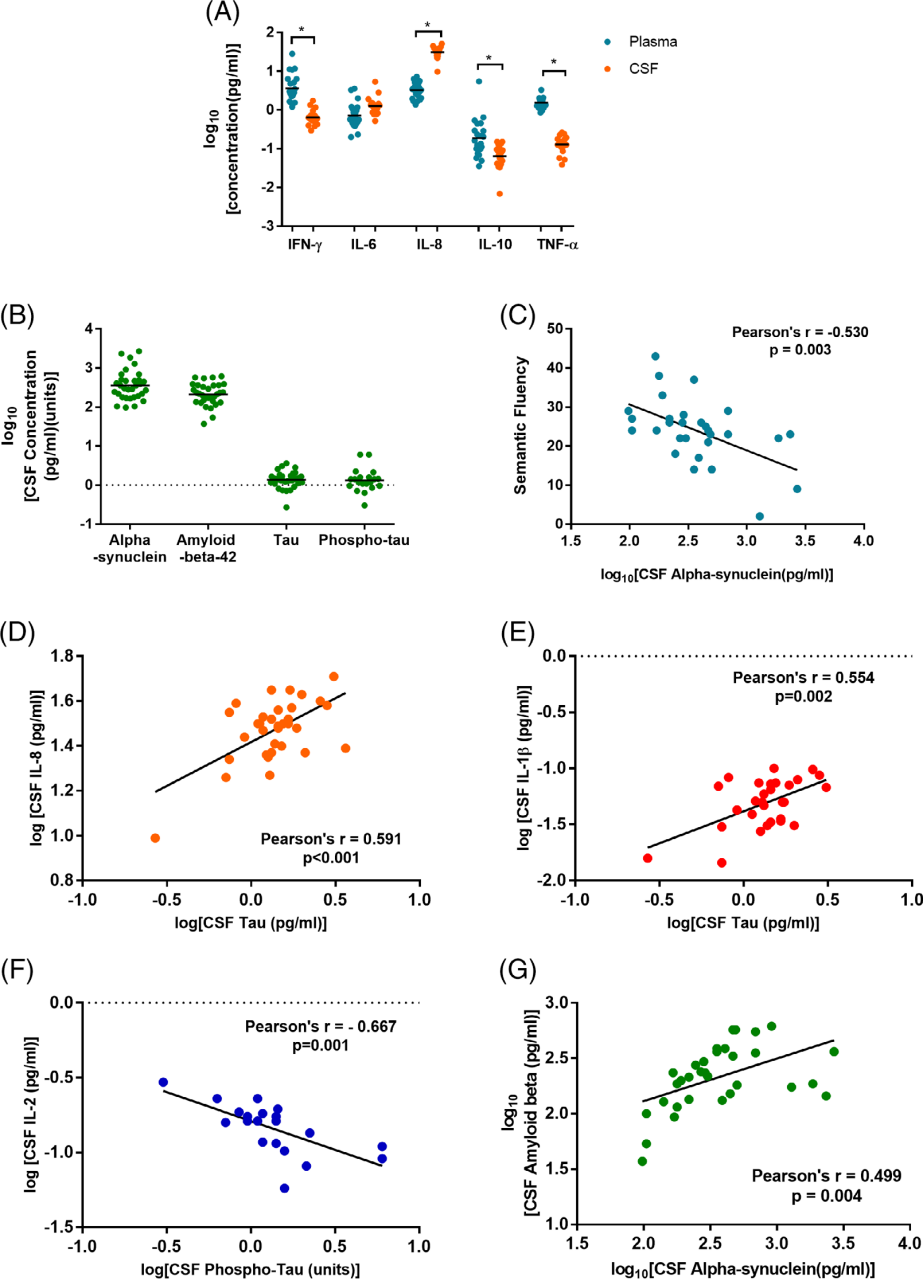
**A:** Paired comparisons of analyzed CSF and plasma cytokines in patients with Parkinson's disease (n = 22). **B:** Mean levels of CSF neurodegenerative proteins in patients with Parkinson's disease (n = 35). **C:** Scatter plot of semantic fluency score versus log_10_CSF α‐synuclein. **D–G:** Graphs demonstrating relationships between CSF markers. CSF, cerebrospinal fluid; IFN, interferon; IL, interleukin; TNF, tumor necrosis factor. [Color figure can be viewed at wileyonlinelibrary.com]

Bivariate analysis of the detected cytokines in both CSF and plasma (IFN‐γ, IL‐6, IL‐8, IL‐10, and TNF‐α) indicated no correlations between CSF and plasma levels in this cohort. Repeated‐measures analysis of variance indicated a significant overall difference between CSF and plasma profiles (*F* = 32.75, *P* < 0.001). Paired comparisons between CSF and plasma values indicated that IL‐8 was significantly higher, whereas IFN‐γ, IL‐10, and TNF‐α were significantly lower in CSF versus plasma following Bonferroni correction for multiple testing (*P* < 0.005; Fig. 1A).

### CSF Cytokines and Neurodegeneration‐Associated Proteins

Both α‐synuclein and amyloid β(1‐42) were the most abundant measured neurodegeneration‐associated proteins in CSF, whereas total tau and phospho‐tau were present at low concentrations (Fig. 1B). Bivariate correlation analyses between all measured CSF neurodegeneration‐associated proteins and CSF cytokines revealed significant relationships between α‐synuclein and amyloid β(1‐42) (Pearson's *r* = 0.499, *P* = 0.004), total tau and IL‐1β (Pearson's *r* = 0.554, *P* = 0.002), total tau and IL‐8 (Pearson's *r* = 0.591, *P* < 0.001), and phospho‐tau and IL‐2 (Pearson's *r* = −0.667, *P* = 0.001; Table S1, Fig. 1D–G).

Multivariate linear regression analyses with each neurodegeneration‐associated protein as the dependent variable confirmed significant positive relationships between tau and IL‐8 (β = 0.779, *P* = 0.001), tau and IL‐1β (β = 0.338, *P* = 0.041), and α‐synuclein and amyloid β(1‐42) (β = 0.605, *P* = 0.010), with age included as covariate (Table 1).

**Table 1:** Linear regression analysis, with log_10_CSF total tau, log_10_CSF phospho‐tau, and log_10_CSF α‐synuclein as the dependent variables

Variable	β Coefficient	Significance	95% Confidence Interval for β	
			Lower	Upper
Dependent variable, log_10_CSF total tau				
Age	0.001	0.869	−0.008	0.009
log_10_CSF IL‐1β	**0.338**	**0.041** *	**0.014**	**0.661**
log_10_CSF IL‐8	**0.779**	**0.001** *	**0.332**	**1.225**
Dependent variable, log_10_CSF phospho‐tau				
Age	0.002	0.874	−0.020	0.024
log_10_CSF IL‐1β	−0.499	0.122	−1.150	0.152
log_10_CSF IL‐2	−0.692	0.104	−1.548	0.164
log_10_CSF TNF‐α	−0.197	0.606	−1.005	0.610
Dependent variable, log_10_CSF α‐synuclein				
Age	0.007	0.455	−0.011	0.024
log_10_CSF amyloid β(1‐42)	**0.595**	**0.011** *	**0.144**	**1.046**

* *P* < 0.05. Bold text indicates results relating to variables with statistical significance (*P* < 0.05).

CSF, cerebrospinal fluid; IL, interleukin; TNF, tumor necrosis factor.

### CSF Markers and Clinical Variables

Bivariate correlation analyses revealed a significant relationship between semantic fluency and α‐synuclein that withstood correction for multiple testing (*r* = −0.53, *P* = 0.003) as well as nominally significant associations (*P* < 0.05) between semantic fluency and IL‐6, ACE‐R and IL‐6, and age and both IFN‐γ and IL‐1β (Table S2). There were no associations with measures of motor severity.

Multivariate linear regression analyses with semantic fluency and ACE‐R score as dependent variables, including age as covariate, confirmed the significant relationship between semantic fluency and α‐synuclein (β = −8.82, *P* = 0.023), but not the associations between ACE‐R and CSF variables (Fig. 1C, Table S3).

## Discussion

This study has demonstrated that the cytokine profile in PD CSF does not relate closely to that seen in the periphery, suggesting that factors within the CNS may play a role in influencing local CNS inflammation. In keeping with this, our data also revealed positive correlations between CSF proinflammatory cytokines (IL‐8, IL‐1β) and CSF total tau in PD. In addition, we have confirmed a positive correlation between CSF α‐synuclein and amyloid β(1‐42) and found a negative correlation between CSF α‐synuclein and semantic fluency—a key clinical predictor of dementia in PD.^10^

Previous studies investigating relationships between CSF and serum/plasma cytokines in PD have reported elevated TNF‐α in CSF compared to serum and correlations between CSF and serum levels of IL‐6, IL‐1β, and IL‐10 in PD and controls.^11^ The different findings compared with our study may reflect variations in cohort demographics, disease stage, and methodology. The lack of CSF–peripheral cytokine correlations in our results suggests that central and peripheral cytokine levels may behave independently of each other and that CSF changes may not simply reflect passive diffusion of circulating cytokines to the CNS. Contributory factors to CSF cytokine levels may include cytokine production from CNS cells (eg, microglia, astrocytes, and neurons), peripheral‐derived immune cells trafficking into the CNS, and active transport across the blood–brain barrier.^12^ Thus, CSF cytokines may better reflect CNS pathology, compared to peripheral cytokines, which may be driven by factors including peripheral α‐synuclein aggregation/pathology, systemic infections/inflammation, and microbial changes (eg, gut microbiome),^13^ with separate relevance to PD and disease progression.^8^

Within PD CSF, the proinflammatory cytokines IL‐1β and IL‐8 correlated positively with total tau, whereas the anti‐inflammatory IL‐2 correlated negatively with phospho‐tau. Potential causal links between tau species and immune changes are unclear, but inflammation has been shown to influence tau production/pathology.^14^ CSF tau/phospho‐tau levels have also been linked to cognitive progression and dementia within PD,^3^ whereas postmortem and genetic studies have connected increased tau pathology and expression with a higher risk of PD cognitive dysfunction/dementia.^15^, ^16^ The current findings linking CSF tau to inflammatory cytokines in PD may therefore be of some relevance in terms of the biological basis of cognitive heterogeneity within PD, regardless of findings in healthy controls. However, control studies will be essential to gain a more complete understanding of the tau–cytokine relationships within the CSF.

The positive correlation between CSF α‐synuclein and amyloid β is consistent with previous studies.^17^ Higher CSF α‐synuclein levels are associated with worse (lower) semantic fluency, which is predictive of the development of PD‐associated dementia.^10^ Although the relationship between CSF α‐synuclein and cognitive function in PD is complex, this result is consistent with previous studies in similar‐stage PD, linking posterior cortical impairment to increased CSF α‐synuclein.^18^ These observations further support the importance of α‐synuclein in the development of PD dementia, as has been demonstrated in postmortem studies.^15^

The limitations of this study include the small sample size, lack of plasma samples in all subjects, and the absence of matched healthy control samples. Furthermore, not all cytokines could be adequately measured in the CSF and plasma using this assay, and higher sensitivity assays may be needed for improved resolution of the low‐level cytokines. However, the assessment of multiple neurodegeneration‐associated proteins and cytokines in simultaneously obtained CSF and plasma samples from a clinically well‐characterized cohort has uniquely allowed interrelationships between these factors to be explored further in PD. Longitudinal assessments of these relationships in larger PD cohorts and matched healthy controls would now be of interest.

### Data Sharing Statement

Data are available upon reasonable request.

## Author Roles

(1) Research project: A. Conception, B. Organization, C. Execution; (2) Statistical Analysis: A. Design, B. Execution, C. Review and Critique; (3) Manuscript: A. Writing of the first draft, B. Review and Critique.

R.S.W.: 1A, 1B, 1C, 2A, 2B, 2C, 3A, 3B

S.F.M.: 1A, 1B, 1C, 2C, 3B

K.F.: 1B, 1C, 2C, 3B

D.P.B.: 1B, 1C, 2C, 3B

R.A.B.: 1A, 1B, 2C, 3B

C.H.W.G.: 1A, 1B, 1C, 2A, 2C, 3B

## Supporting information


**Table S1** **‐** Summary of significant correlations between neurodegeneration‐associated proteins and cytokines. * = Remained significant post Bonferroni correction.
**Table S2 –** Significant correlations between clinical variables and CSF markers on bivariate analysis. * = Remained significant post Bonferroni correction.
**Table S3 ‐** Linear Regression with ACE‐R score and semantic fluency as the dependent variables. *p < 0.05.
