# Supplementary material for: Cerebrospinal Fluid Cytokines and Neurodegeneration‐Associated Proteins in Parkinson's Disease
Source: Mov Disord. 2020 Mar 4;35(6):1062–6. doi: 10.1002/mds.28015 (PMC8629119; doi:10.1002/mds.28015)
Supplement: Supplementary file 1 — Table S1 ‐ Summary of significant correlations between neurodegeneration‐associated proteins and cytokines. * = Remained significant post Bonferroni correction. Table S2 – Significant correlations between clinical variables and CSF markers on bivariate analysis. * = Remained significant post Bonferroni correction. Table S3 ‐ Linear Regression with ACE‐R score and semantic fluency as the dependent variables. *p < 0.05. [file MDS-35-1062-s001.docx]

**SUPPLEMENTARY DATA**

| **Factors (log_10_)** | **CSF cytokine or neurodegeneration-associated protein** | **Correlation**  **(Pearson’s r)** | **Significance (p)** |
| --- | --- | --- | --- |
| CSF alpha-synuclein | log_10_ CSF amyloid beta | 0.499 | 0.004* |
| CSF amyloid beta(1-42) | log_10_ CSF IL-4 | -0.731 | 0.025 |
| CSF tau | log_10_CSF IL-1β | 0.554 | 0.002* |
|  | log_10_CSF IL-8 | 0.591 | <0.001* |
| CSF phospho-tau | log_10_CSF IL-1β | -0.537 | 0.022 |
|  | log_10_CSF IL-2 | -0.667 | 0.001* |
|  | log_10_CSF TNF-α | -0.456 | 0.043 |

***Table S1 -*** *Summary of significant correlations between neurodegeneration-associated proteins and cytokines. *=Remained significant post Bonferroni correction.*

| **Clinical Variable** | **CSF marker** | **Correlation**  **(Pearson’s r)** | **Significance (p)** |
| --- | --- | --- | --- |
| Semantic Fluency | log_10_CSF IL-6 | 0.418 | 0.021 |
|  | log_10_ CSF alpha-synuclein | -0.530 | 0.003* |
| ACE-R score | log_10_CSF IL-6 | 0.366 | 0.043 |
| Age | log_10_CSF IFN-γ | -0.410 | 0.027 |
|  | log_10_CSF IL-1β | -0.369 | 0.049 |

***Table S2 –*** *Significant correlations between clinical variables and CSF markers on bivariate analysis. * = Remained significant post Bonferroni correction.*

| **Variable** | **Beta Coefficient (B)** | **Significance** | **95% Confidence Interval for B** | |
| --- | --- | --- | --- | --- |
|  |  |  | **Lower** | **Upper** |
| **Dependent variable – ACE-R** |  |  |  |  |
| Age | -0.408 | 0.058 | -0.830 | 0.014 |
| log_10_CSF IL-6 | 14.006 | 0.080 | -1.784 | 29.797 |
|  |  |  |  |  |
| **Dependent variable – Semantic fluency** |  |  |  |  |
| Age | -0.273 | 0.215 | -0.714 | 0.169 |
| log_10_CSF IL-6 | 10.184 | 0.129 | -3.178 | 23.546 |
| **log_10_CSF alpha-synuclein** | **-8.825** | **0.023*** | **-16.346** | **-1.304** |

***Table S3 -*** *Linear Regression with ACE-R score and semantic fluency as the dependent variables. *p<0.05.*
